# Neurodegeneration in a *Drosophila* model of adrenoleukodystrophy: the roles of the Bubblegum and Double bubble acyl-CoA synthetases

**DOI:** 10.1242/dmm.022244

**Published:** 2016-04-01

**Authors:** Anna Sivachenko, Hannah B. Gordon, Suzanne S. Kimball, Erin J. Gavin, Joshua L. Bonkowsky, Anthea Letsou

**Affiliations:** 1 Department of Human Genetics, University of Utah, Salt Lake City, UT 84112, USA; 2 Department of Pediatrics, University of Utah, Salt Lake City, UT 84112, USA

**Keywords:** *Drosophila*, Neurodegeneration, Acyl-CoA synthetase, VLCFA, *Bubblegum*, *Double bubble*, *SLC27a6*

## Abstract

Debilitating neurodegenerative conditions with metabolic origins affect millions of individuals worldwide. Still, for most of these neurometabolic disorders there are neither cures nor disease-modifying therapies, and novel animal models are needed for elucidation of disease pathology and identification of potential therapeutic agents. To date, metabolic neurodegenerative disease has been modeled in animals with only limited success, in part because existing models constitute analyses of single mutants and have thus overlooked potential redundancy within metabolic gene pathways associated with disease. Here, we present the first analysis of a very-long-chain acyl-CoA synthetase (ACS) double mutant. We show that the *Drosophila bubblegum* (*bgm*) and *double bubble* (*dbb*) genes have overlapping functions, and that the consequences of double knockout of both *bubblegum* and *double bubble* in the fly brain are profound, affecting behavior and brain morphology, and providing the best paradigm to date for an animal model of adrenoleukodystrophy (ALD), a fatal childhood neurodegenerative disease associated with the accumulation of very-long-chain fatty acids. Using this more fully penetrant model of disease to interrogate brain morphology at the level of electron microscopy, we show that dysregulation of fatty acid metabolism via disruption of ACS function *in vivo* is causal of neurodegenerative pathologies that are evident in both neuronal cells and their supporting cell populations, and leads ultimately to lytic cell death in affected areas of the brain. Finally, in an extension of our model system to the study of human disease, we describe our identification of an individual with leukodystrophy who harbors a rare mutation in *SLC27a6* (encoding a very-long-chain ACS), a human homolog of *bgm* and *dbb*.

## Abstract (precis)

***Drosophila* Collection:** A new *Drosophila* model of ALD reveals dysregulation of fatty acid metabolism as causal of neurodegenerative pathologies and has led to the identification of a new candidate gene for ALD in humans.

## INTRODUCTION

### Adrenoleukodystrophy

Adrenoleukodystrophy (ALD) is a rare and oftentimes fatal progressive neurodegenerative disease. The most common form of the disease is X-linked (X-ALD), with an estimated incidence of 1:17,000 in all ethnic groups. X-ALD is a clinically heterogeneous disorder, exhibiting incomplete penetrance and variable expressivity (Moser et al., 2005).

The most severe form of X-ALD, affecting ∼40% of all affected individuals, is cerebral ALD. Demyelination in the central nervous system (CNS) constitutes the major burden of cerebral ALD, with the disorder most frequently diagnosed as progressive neurological dysfunction in previously healthy boys between the ages of 4 and 8 years old. A less severe, but nonetheless debilitating, form of the disease [adrenomyeloneuropathy (AMN)] occurs in ∼40% of individuals with X-ALD. In individuals with AMN, demyelination is confined (at least initially) to the peripheral nervous system (PNS). Individuals with AMN present in the second to fourth decade of life with impotence, sphincter dysfunction and slowly progressive paraparesis. About 10% of individuals with X-ALD have adrenal insufficiency without nervous system involvement; a similar small number of males remain entirely asymptomatic. It is notable that wide phenotypic variability is observed within kindreds segregating the same mutation; thus, environmental and/or genetic factors must play substantial roles as disease modifiers (Moser, 1997).

### Function of the ATP-binding-cassette transporters in ALD

**Fig. 1.**
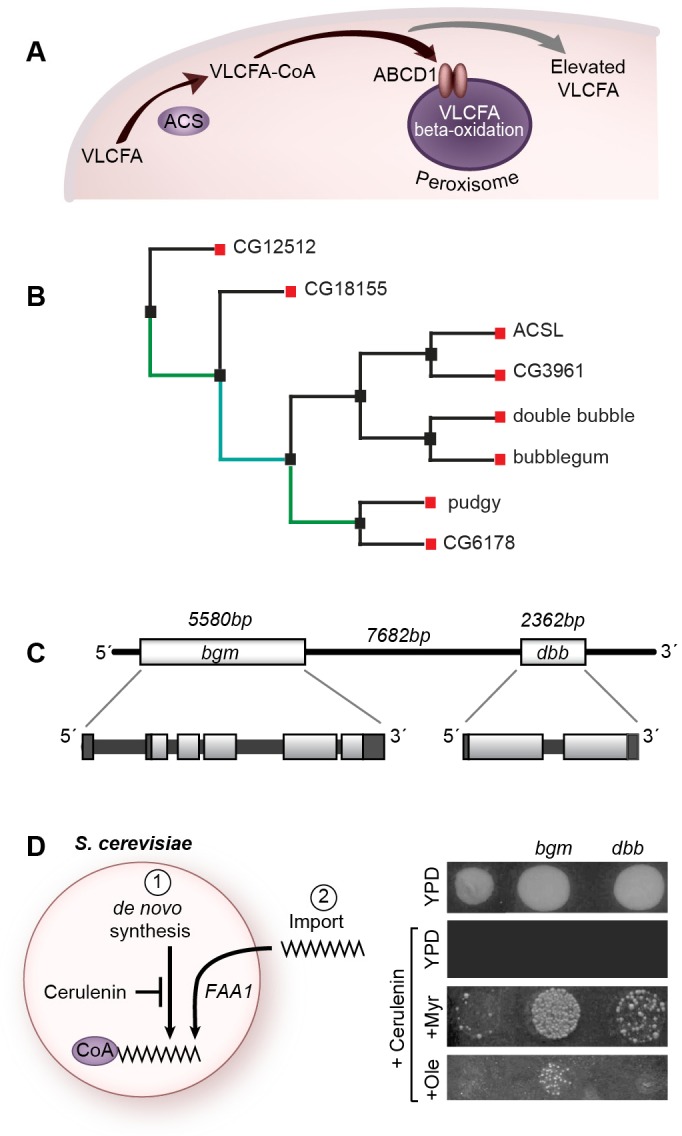
***bgm* and *dbb* are duplicated genes that encode bona fide acyl-CoA synthetases.** (A) Enzymology of VLCFA degradation in peroxisomes. When transport to the peroxisome is blocked by mutation of either the synthetase (ACSL) or the transporter (ABCD1), which is shown here as a dimer (oval pair) resident in the peroxisomal membrane, VLCFAs accumulate intracellularly (gray arrow). (B) A phylogenetic tree of all annotated mono-functioning acyl-CoA synthetase proteins in *D. melanogaster*. The tree was made using the software PhylOgenetic Web Repeater (POWER) by aligning amino acid sequences. Standard conditions were used, with the exception of the following: no outgroup was applied and bootstrapping was used to determine the best-fit tree. Colors indicate the number of times that line was established out of 100 assembled phylogenetic trees (bootstrapping). Teal indicates between 40-60 times, and green indicates between 60-80 times. (C) Organization of the *bubblegum* (*bgm*) and *double bubble* (*dbb*) genes on *Drosophila* chromosome 2. (D) Cerulenin renders yeast auxotrophic and dependent on imported fatty acids and the activity of *FAA1*. Both *bgm* and *dbb* restore growth to *FAA1-*deficient yeast when myristate is added to the growth medium. Only *bgm* complements *FAA1* in the presence of oleate. YPD, yeast peptone dextrose.

The gene responsible for X-ALD has been cloned and shown to encode a peroxisomal half ATP-binding-cassette transporter [ATP-binding cassette, sub-family D (ALD), member 1 (*ABCD1*); Mosser et al., 1993]. The 745-amino-acid ABCD1 protein [also known as adrenoleukodystrophy protein (ALDP)] localizes to the peroxisomal membrane, where, like other members of the ABC (ATP-binding cassette) transporter family, it functions to transport very-long-chain fatty acids (VLCFAs; 22 or more carbons) via their acyl-CoA esters into peroxisomes where they are degraded by β-oxidation (Fig. 1A). Indeed, accumulation of saturated VLCFAs is a known biochemical hallmark of X-ALD (Igarashi et al., 1976). All individuals with X-ALD, including asymptomatic carriers, show elevated levels of VLCFAs, in particular tetracosanoic acid (C24:0) and hexacosanoic acid (C26:0) in the plasma, brain and adrenal glands. There is more recent evidence for the additional accumulation of monounsaturated VLCFAs in plasma and fibroblasts of individuals with X-ALD (Kemp et al., 2005). This finding has especially important consequences with respect to treatment of individuals with the highly controversial Lorenzo's oil, itself a mixture of monounsaturated VLCFAs.

### Function of very-long-chain acyl-CoA synthetases in ALD

Acyl-CoA synthetases (ACSs), which function immediately upstream of ABC transporters in effecting peroxisomal degradation of fatty acids, have also been implicated in ALD disease pathology (see Fig. 1A). Chemically reactive fatty acyl-CoA esters, which are derived from their chemically inactive precursors (free fatty acids) in a two-step process, serve as substrate for the ABCD1 transporter. It is the second step of the fatty acid esterification process that is catalyzed by ACS. There are four recognized enzyme families with ACS activity: those activating short (2-5 carbons), medium (6-12 carbons), long (13-21 carbons) and very long (≥22 carbons) fatty acid substrates. Although families are defined by substrate specificities, it is common for these to overlap between families, particularly with respect to the long- and very-long-chain substrates (Steinberg et al., 1999a,b; Watkins et al., 2007; Watkins, 2008; Lu et al., 2009). The essential role of ACSs in fatty acid metabolism is supported by their high degree of conservation in organisms ranging from bacteria to humans. ACSs are defined by two highly conserved functional domains: the ten-amino-acid AMP-binding domain and the 35-amino-acid signature motif that plays an integral role in determining substrate specificity (Watkins, 2008).

### Animal models of ALD

Investigators have long sought to resolve the clinical heterogeneity of ALD in animal models, thus far, however, with only very limited success. Phenotypes associated with knockout of *ABCD1* in mice are dependent upon genetic background. In one *ABCD1-*knockout mouse model of X-ALD, mice experience a normal lifespan and exhibit no signs of degeneration in either the CNS or PNS. The same knockout in a different genetic background, however, does lead (albeit at low frequency) to accumulation of VLCFAs and development of AMN-type symptoms (Forss-Petter et al., 1997; Lu et al., 1997). With regard to ACS loss of function, neither neurological defects nor VLCFA accumulation is detected in *VLCS-1* (VLCFA acyl-CoA synthetase)-knockout mice (Heinzer et al., 2003).

Data obtained in a fly model of ACS function are more encouraging, because mutation of *bubblegum* (*bgm*), the gene encoding a *Drosophila* long-chain ACS (ACSL) with predicted long- and very-long-chain substrate specificities, results in an autosomal recessive neurodegenerative phenotype (Min and Benzer, 1999). *bgm* homozygotes exhibit impaired vision and a ‘bubbly’ optic lobe, phenotypes resulting from a loss of nervous tissue. The documented *bgm-*associated biochemical and neurodegenerative abnormalities are subtle, being limited to moderate accumulation of two saturated VLCFAs, and minimal disorganization and degeneration of the optic lobe. *bgm* flies have, therefore, not been embraced as a strong animal model for ALD.

Until now, what none of the ABC transporter or ACS animal studies (neither fly nor mouse) have adequately contemplated is the considerable potential for redundancy within these gene families. With regard to the latter, although original classifications of the ACSs were based solely on the fatty acid chain lengths of their substrates, more recent studies point to overlapping substrate specificities (Watkins et al., 2007; Watkins, 2008). Indeed, having confirmed that the *bgm* allele used by Min and Benzer (1999) is a null, we show here that a far more profound phenotype, and thus a better ALD disease model, is found in double-mutant animals harboring mutations not only in the *bgm-*encoded ACSL, but also in its homolog, the *double bubble* (*dbb*)-encoded ACSL.

As described in this article, our focus on the similarities between *bgm dbb* and ALD phenotypes provide new insights into defects associated with the accumulation of VLCFAs. In particular, we show that defects in VLCFA metabolism affect neurons as well as their supporting cells, and lead to widespread cell losses in the fly brain. In addition, our detection of inclusions in *bgm dbb* mutant flies augurs an attractive diagnostic tool. Underscoring the relevance of ACSL mutations to ALD phenotypes, we also describe our identification of a young boy with an undiagnosed leukodystrophy who harbors a mutation in *SLC27a6*, encoding a very-long-chain ACS with homology to Bgm and Dbb (Steinberg et al., 1999a,b, 2000).

## RESULTS

### *bgm* and *dbb* encode duplicated *Drosophila* acyl-CoA synthetases

Our studies have their foundation in the postulate that ACSs in *Drosophila* have overlapping and hence sometimes redundant function. In this regard, there are multiple ACSs in all species. In *Drosophila*, in addition to *bgm*, there are seven genes encoding fatty acid ACSs (Fig. 1B). *bgm* and *CG4500* [which, based on its genetically defined loss-of-function phenotype, is designated here as *double bubble* (*dbb*)] are more closely related to one another than they are to the other members of their family. *bgm* and *dbb* share 43% identity and 62% similarity at the amino acid level. Furthermore, *bgm* and *dbb* map immediately adjacent to one another in the *Drosophila* genome (7.7 kb apart; Fig. 1C). The *bgm* and *dbb* genes belong to the evolutionarily well-conserved Bgm family of ACSLs. The human homolog (hBgm) is 41% identical (61% similar) to *Drosophila* Bgm, and 37% identical (55% similar) to *Drosophila* Dbb (Fig. S1).

### The *bgm* and *dbb* gene products retain their predicted acyl-CoA synthetase activity

To test whether the *bgm* and *dbb* gene products retain their predicted ACS activity, we assessed *bgm* and *dbb* activity in a yeast heterologous system. Expression of either the *bgm* or *dbb Drosophila* cDNA in a *Saccharomyces* *cerevisiae* strain that is null for Faa1p (the major ACSL in yeast) restored strain viability in the presence of the *de novo* fatty acid synthesis inhibitor cerulenin when myristate was added to the culture medium; only *bgm*, however, rescued in the presence of oleate (Fig. 1D). (Both myristate and oleate represent long-chain fatty acids, C14:0 and C18:1, respectively.) Thus, although both *bgm* and *dbb* retain ACS activity, their substrate specificities are overlapping, but not identical.

### Knocking out *bgm* and *dbb* gene functions

**Fig. 2.**
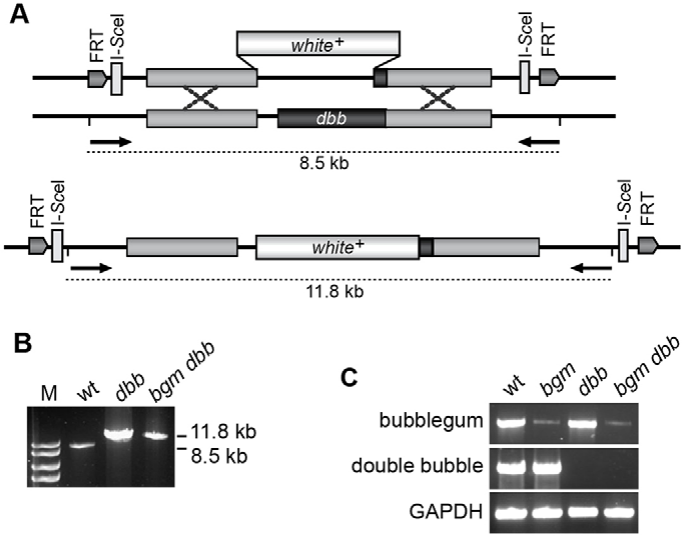
**Generation of *dbb* and *bgm dbb* mutant animals.** (A) Schema showing the ends-out gene-targeting strategy employed for generation of *dbb* single- and *bgm dbb* double-mutant animals, as well as the positions of primers used for analytical PCR. (B) Verification of the putative *dbb^1^* and *bgm^1^ dbb^1^* alleles by PCR. M denotes the marker lane and includes bands corresponding to 10, 8, 6 and 5 kb lengths. (C) Verification of loss of *dbb* expression in *dbb^1^* and *bgm^1^ dbb^1^* knockout animals by RT-PCR. wt, wild type.

Extensive similarity between Bgm and Dbb at the protein level, as well as their overlapping substrate specificities, led us to speculate that the *bgm-* and *dbb*-encoded ACSL functions are redundant. We tested this hypothesis in genetic analyses of single and double mutants. We used the previously characterized *bgm^1^* null allele (a P{lacW} insertion in the gene's first intron) for our study, but turned to targeted knockout methods to generate the *dbb^1^* null allele and the *bgm^1^ dbb^1^* double-null mutant (Fig. 2A). PCR analysis allowed discrimination of wild-type and mutated loci based on a 3.3-kb fragment differential at the *dbb^1^* locus (Fig. 2B). Measurement of transcript levels in wild-type and mutant animals revealed traces of *bgm* mRNA in the previously characterized *bgm^1^* null background (Min and Benzer, 1999), but no detectable *dbb* mRNA in the genetically engineered *dbb^1^* null (Fig. 2C).

### *bgm* and *dbb* function redundantly in the adult CNS

To study the functional relationship between *bgm* and its homolog *dbb* in the maintenance of adult CNS integrity, we analyzed brain morphologies in wild-type flies, and in single- and double-mutant flies. For histological studies, heads from 18-day-old wild-type (Canton-S) and mutant (*bgm^1^*, *dbb^1^* and *bgm^1^ dbb^1^*) flies were embedded in paraffin, stained with hematoxylin and eosin (H&E), and scored blindly under a light microscope for the presence of: (a) optic ganglion (laminal) holes with diameters greater than 3 μm, (b) retinal disorganization, and (c) fenestrated membrane loss. Whereas the optic ganglion phenotype was quantified by hole number, the degree of retinal degeneration was scored qualitatively as 1 for normal appearance, 2 for mild tissue loss, 3 for moderate degeneration and 4 for severe degeneration. For the assessment of fenestrated membrane morphologies, we scored images from 1 (normal) to 5 (very severely damaged), taking into account membrane thickness, the presence of gaps, displacement of retinal cells into the optic lobe and irregularity of the basement membrane. To obtain the mean score for each animal, we analyzed at least eight hemibrain sections from the same fly. Data from five flies per genotype were analyzed by ANOVA and Welch two-sample *t*-tests.

**Fig. 3.**
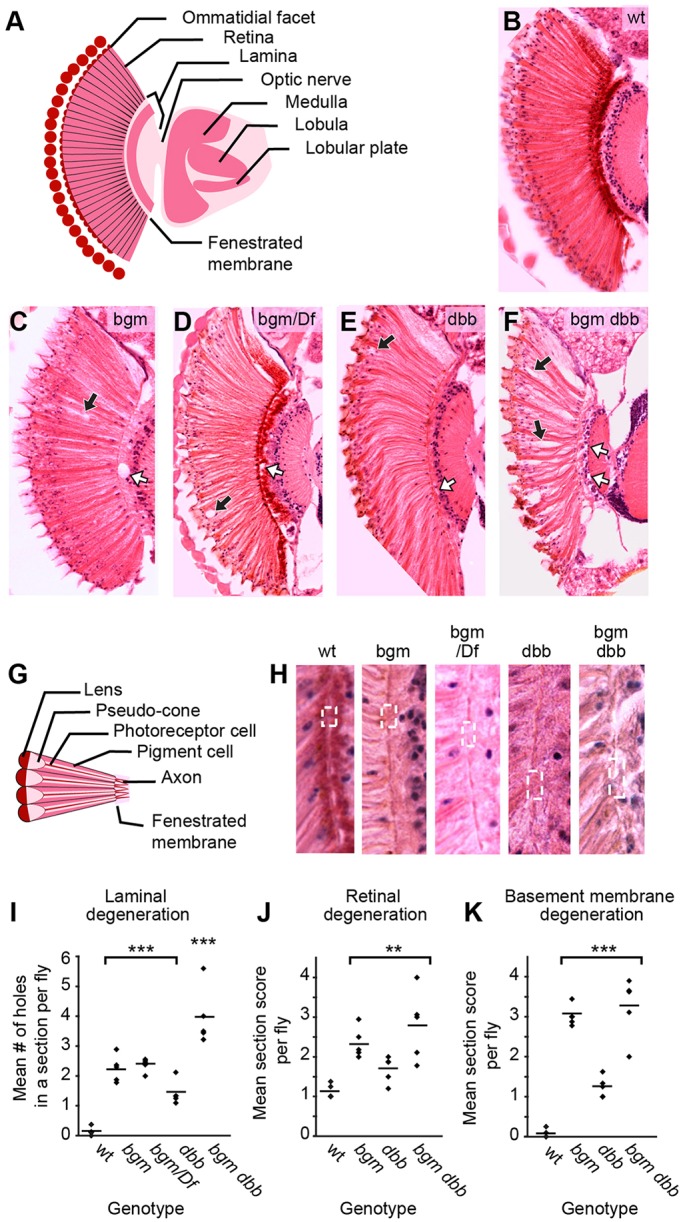
**Degeneration of the CNS in acyl-CoA synthetase mutants.** (A) Labeled schematization of a *Drosophila* brain horizontal section. (B-F) H&E-stained brain sections representative of wild-type, *bgm^1^*, *bgm^1^/Df(2L)b87e25*, *dbb^1^* and *bgm^1^ dbb^1^* animals, respectively. In C-F, retinal degeneration is marked by black arrows and laminal degeneration by white arrows. (G) Labeled schematization of a horizontal section of the *Drosophila* retina. (H) Magnified fenestrated membranes from animals shown in B-F. Note the enlarged portals (dashed boxes, containing in the case of the *bgm dbb* double mutant displaced retinal nuclei). (I-K) Degeneration in (I) lamina and (J) retina, as well as (K) defects in the fenestrated basement membrane, were quantified. For these plots, data obtained from analysis of H&E brain sections were analyzed by ANOVA and by the Welch two-sample *t*-test. Each point represents the mean score per animal; horizontal lines represent the mean score per genotype. Statistical significance determined by ANOVA followed by Student's *t*-test: ***P<0.001, **P≤0.015. wt, wild type.

Although fully viable, *dbb^1^* and *bgm^1^ dbb^1^* adults exhibited clear CNS defects, suggesting that *dbb* (like *bgm*) is required for the maintenance of adult nervous system tissue (Fig. 3A-F). We documented laminal holes in *bgm^1^*, *bgm^1^/Df(bgm)*, *dbb^1^* and *bgm^1^ dbb^1^* 18-day-old mutant flies but not in age-matched wild-type controls (Fig. 3I), nor in newly eclosed mutants as was expected from previous reports (Min and Benzer, 1999). We observed no significant differences in the degree of degeneration of the lamina of age-matched *bgm^1^* homozygotes and *bgm^1^*/*Df(bgm)* transheterozygotes (*P*=0.13), thereby genetically establishing the amorphic character of the *bgm^1^* allele. Statistical analyses revealed differences in hole numbers between single mutants and wild types to be highly significant (*P*≤0.001); thus, each gene is individually required for neuronal maintenance. Holes were most abundant in *bgm^1^ dbb^1^* double mutants (*P<*0.0002 for comparisons both to wild types and single mutants). Thus, these data indicate: (1) that, in the laminal region of the brain, the *bgm* and *dbb* genes function redundantly, and (2) that the double-mutant animal is fully penetrant and provides a more powerful resource (than either of the single mutants alone) for determining the effects of fatty acid dysregulation in neurodegeneration.

Histological examinations of H&E-stained brain sections revealed that, in addition to laminal degeneration, which had been documented although not previously quantified for *bgm* (Min and Benzer, 1999), both single and double mutants also exhibit phenotypes not previously described. In this regard, we observed retinal degeneration, as well as thinning and irregularity of the fenestrated membrane – the structure defining the anatomic border between the retina and lamina – in *bgm^1^*, *dbb^1^* and *bgm^1^ dbb^1^* mutant animals. That the atypical fenestrated membrane in ACSL mutants is functionally encumbered is most evident in double-mutant animals where retinal nuclei can routinely be seen crossing from the retina into the lamina (Fig. 3G,H)*.* In quantifying retinal and fenestrated membrane defects (Fig. 3J,K), we found that dysmorphologies are stronger in the *bgm^1^* mutant than in the *dbb^1^* mutant (*P*≤0.02), although, in comparisons to wild type, it was clear that *dbb* does contribute in a significant fashion to retinal and fenestrated membrane structure (*P*=0.015 and *P*=0.00017, respectively). Our comparisons of retinal and fenestrated membrane degeneration in *bgm^1^* and *bgm^1^ dbb^1^* mutant animals did not reveal statistically significant differences between the two (*P*=0.34 and *P*=0.54), but, as is true for ALD in humans, wide phenotypic variability was observed in double mutants, and the highest levels of degeneration were documented in double mutants only. Taken together, comparisons of wild-type and mutant retinal and laminal brain sections demonstrated that: (1) loss of the *bgm-* and/or *dbb-*encoded ACSL results in statistically significant levels of nervous system impairment in all regions tested, and (2) cooperativity between the two genes varies by brain region, which is indicative of both overlapping (laminal) and cell-selective (retinal) outcomes for this gene pair.

### Mutations in *bgm* and *dbb* affect lipid- and membrane-rich pigment cells

**Fig. 4.**
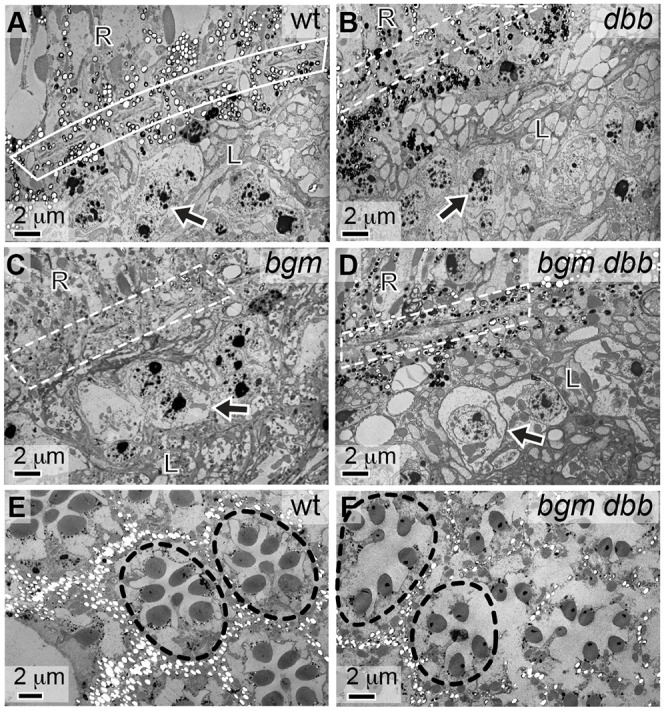
**Neuronal death and pigment cell defects in Acyl-CoA synthetase mutants.** Ultrastructural analysis of heads from 18-day-old (A) wild-type (wt), (B) *dbb^1^*, (C) *bgm^1^* and (D) *bgm^1^ dbb^1^* flies. In wild-type animals, the fenestrated membrane appears as membrane with pores (boxed in panel A). Only a thinned or barely visible membrane is visible in mutant animals (boxed in panels B-D). Also, lytic death of monopolar neurons is evident in the lamina of mutant, but not wild-type, animals. In A-D, monopolar neurons are indicated by arrows; R denotes the retinal domain; L denotes the laminal domain. Ultrastructural analysis of retinas from 18-day-old (E) wild-type and (F) *bgm^1^ dbb^1^* flies. Loss of the ommatidial structure (outlined) in the *bgm dbb* double-mutant retina is correlated with loss of secondary pigment cells; these surround each ommatidial cluster of photoreceptor neurons and in these images appear as empty white structures due to pigment loss associated with processing (Cagan and Ready, 1989; Wolff and Ready, 1991).

To gather insight into the cellular origin of the *bgm* and *dbb* neurodegenerative phenotypes, we next turned to transmission electron microscopy (TEM). We focused first on the fenestrated membrane, which forms the physical and electrical barrier between the eye and the brain. As suggested by our initial H&E-based analysis, we observed structural abnormalities of the fenestrated membrane in ASCL mutants (Fig. 4A-D). Ultrastructural imaging of wild-type and mutant eyes from 18-day-old adults further revealed retinal holes, disarray in the normal hexagonal pattern of ommatidial structure and, perhaps most tellingly, loss of pigment cells (the non-neuronal supporting cells that surround and insulate single retinal ommatidial clusters) in mutants (Fig. 4E,F).

The most parsimonious explanation for the fenestrated membrane defects that we observed in ACSL mutant flies is that they arise after patterning of the *Drosophila* eye is complete. Although fenestrated membrane abnormalities have been attributed – in other mutant backgrounds – to defects in cell patterning during imaginal stages of *Drosophila* eye development, this class of patterning mutant reveals itself as a rough-eye phenotype in adults, readily observed either by scanning electron microscopy or by visual examination (Coyle-Thompson and Banerjee, 1993). Importantly, we have looked for the rough-eye phenotype (by visual inspection; *n*>100/genotype), but have yet to observe it in any of our ACSL mutants. In addition, whereas ommatidial disarray and degeneration are clear in aged animals, normal ommatidial patterning was always observed at the time of eclosion (Fig. S2).

**Fig. 5.**
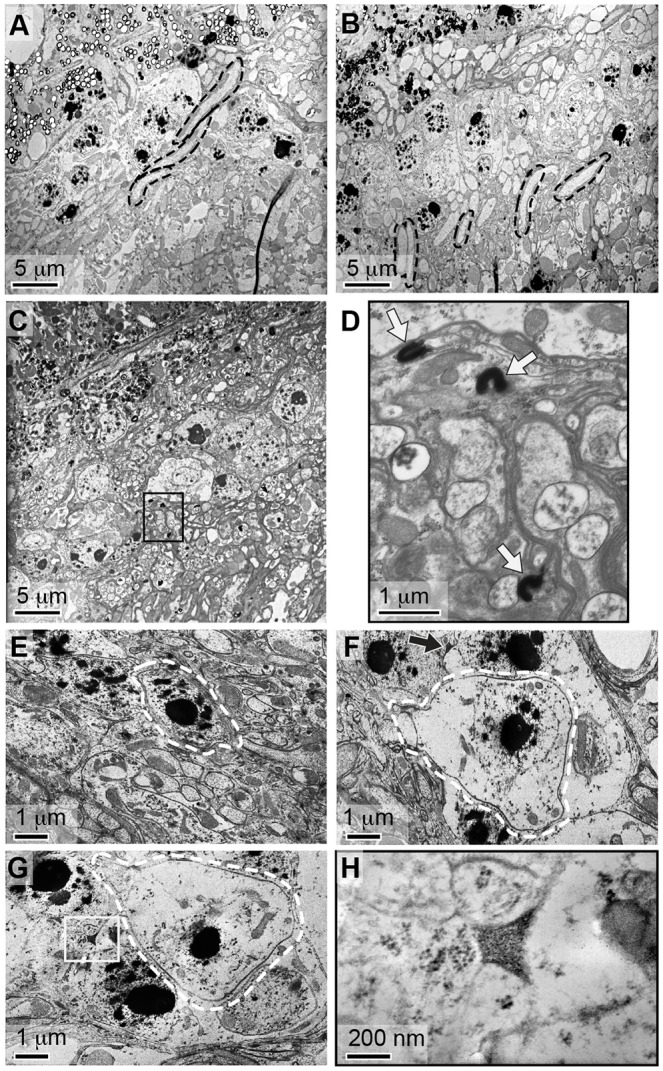
**Neurodegeneration is associated with laminal deposits.** Ultrastructural analyses of lamina in (A) wild-type, (B) *dbb^1^* and (C) *bgm^1^* mutant animals reveal the lamina of mutants to be highly disorganized. Note the parallel organization of photoreceptor axons in wild-type brains, and their misorientation in *dbb* mutants (outlined in black in A and B). These structures are absent from *bgm* mutants in which densely stained inclusions are abundant (e.g. boxed region in C). (D) The boxed region from panel C was imaged at higher magnification, and inclusions are indicated with arrows. (E) In wild-type animals, monopolar neurons (outlined in white) are distinguished by their nucleolar content. In *bgm dbb* double mutants (F,G), however, monopolar neurons are distinguished by cytoplasmic swelling and lytic cell death. Lysing monopolar neurons are oftentimes found in association with extracellular precipitates (arrow in F and boxed in G). (H) The precipitate from panel G was imaged at higher magnification, revealing it to be granular and thus structurally identical to previously described fatty acid precipitates.

Next, we considered the lamina. Although histological studies reported here and elsewhere (Min and Benzer, 1999) revealed gross degenerative phenotypes in ACSL mutants, our TEM studies more clearly revealed the associated cellular abnormalities. Particularly notable in this regard is neuronal disorganization (Fig. 5A,B), a phenotype that might result from defects in axonal pathfinding occurring in conjunction with abnormalities in fenestrated membrane portals. Moreover, inclusions like those observed in neurodegenerative diseases appeared frequently in the lamina of *bgm^1^* mutant animals (Fig. 5C,D). Assays with the neutral lipid stains Oil Red O and Sudan black reveal no accumulations in the brains of mutants in comparison to wild type (Fig. S3). Nonetheless, it is tempting to speculate that inclusions in mutants could be a direct consequence of accumulating VLCFAs, because polyunsaturated VLCFAs induce formation of proteinaceous inclusions in a cell-culture model of Parkinson's disease (Assayag et al., 2007). Ultrastructural studies capture lytic cell death in monopolar neurons, here visualized by cell swelling and cytoplasmic clearing (Fig. 5E-G; see also Fig. 4C,D). Finally, accumulating granular material in the proximity of dying neurons is consistent with the view that monopolar neuron death might be due, at least in part, to toxic cellular inclusions of unknown content (Fig. 5H).

### ACSL mutant flies recapitulate the full array of human ALD phenotypes

**Fig. 6.**
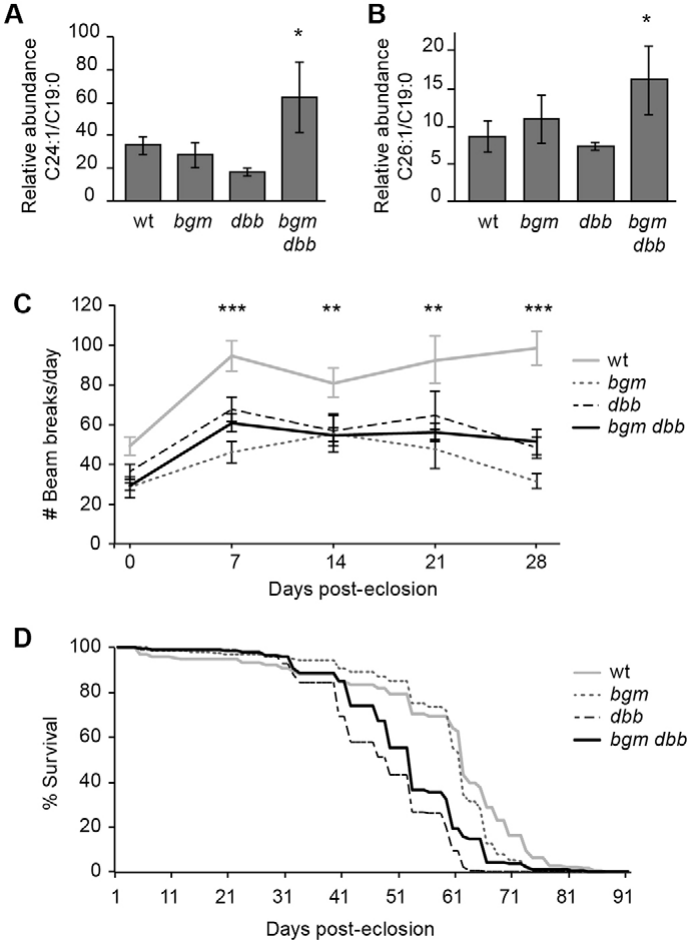
***bgm dbb* mutants recapitulate adrenoleukodystrophy (ALD) phenotypes.** Gas chromatography-mass spectrometry analysis (GC-MS) of (A) C24:1 and (B) C26:1 in *bgm dbb* mutant heads; *P=0.04. C19:0 is an experimentally spiked standard and is used to normalize samples. (C) Locomotor activity in wild-type and mutant flies. (A-C) Student's *t*-test, with s.e.m. shown; *P<0.05, **P<0.01, ***P<0.001. (D) Kaplan–Meir survival estimates for wild-type and mutant flies (*n*≥150/genotype). wt, wild type.

Having established gross anatomical phenotypes in brains of *bgm^1^*, *dbb^1^* and *bgm^1^ dbb^1^* adults, we sought to determine whether these defects are associated with significant changes in fatty acid levels and/or behavioral abnormalities, features that have remained statistically undocumented in previous loss-of-function studies in ALD mouse and fly models. Gas chromatography-mass spectrometry (GC-MS) profiling of all fatty acid species of chain length C12-C26 in wild-type and ACSL single- and double-mutant flies revealed only two statistically significant differences: twofold increases of the C24:1 and C26:1 monounsaturated VLCFAs in *bgm^1^ dbb^1^* double-mutant animals (Fig. 6A,B). Moreover, in tests of locomotor activity, all mutants showed an inability to match wild-type activity levels, as evidenced by a decrease in the average number of beam breaks per animal within the 2-h period before and after light change. Statistically significant differences in activity levels were not evident at eclosion but were at or near maximal levels by 7 days post-eclosion (Fig. 6C). Notably, and in contrast to the enhanced double-mutant retinal defect, single and double mutants displayed analogous locomotor defects. Although these data are consistent with the idea that decreased locomotion is a secondary consequence of neurodegeneration, it might also be true that *bgm* and *dbb* functions are pleiotropic in the adult fly, affecting the two systems independently.

When mutations in *Drosophila* genes are associated with neurodegeneration, phenotypes oftentimes extend to lifespan reduction (Greenspan and Dierick, 2004), and indeed this has been reported for *bgm* (Min and Benzer, 1999). In contrast to previous reports, we found that loss of *bgm* does not shorten lifespan and that loss of *dbb* only shortens life by about 10% (Fig. 6D). The difference between results from our *bgm* lifespan study and those reported previously is likely attributable to differences in experimental protocol. For the lifespan analyses reported here, we employed congenic recombinant inbred strains on an isogenic Canton-S background. Similar to our observations, lifespan changes associated with a mutation in the *Drosophila* gene *Indy*, coding for a Krebs cycle intermediate transporter, were abolished upon backcrossing into a different genetic background (Toivonen et al., 2007).

### A leukodystrophy patient harbors a mutation in *SLC27a6*, encoding a human acyl-CoA synthetase

**Fig. 7.**
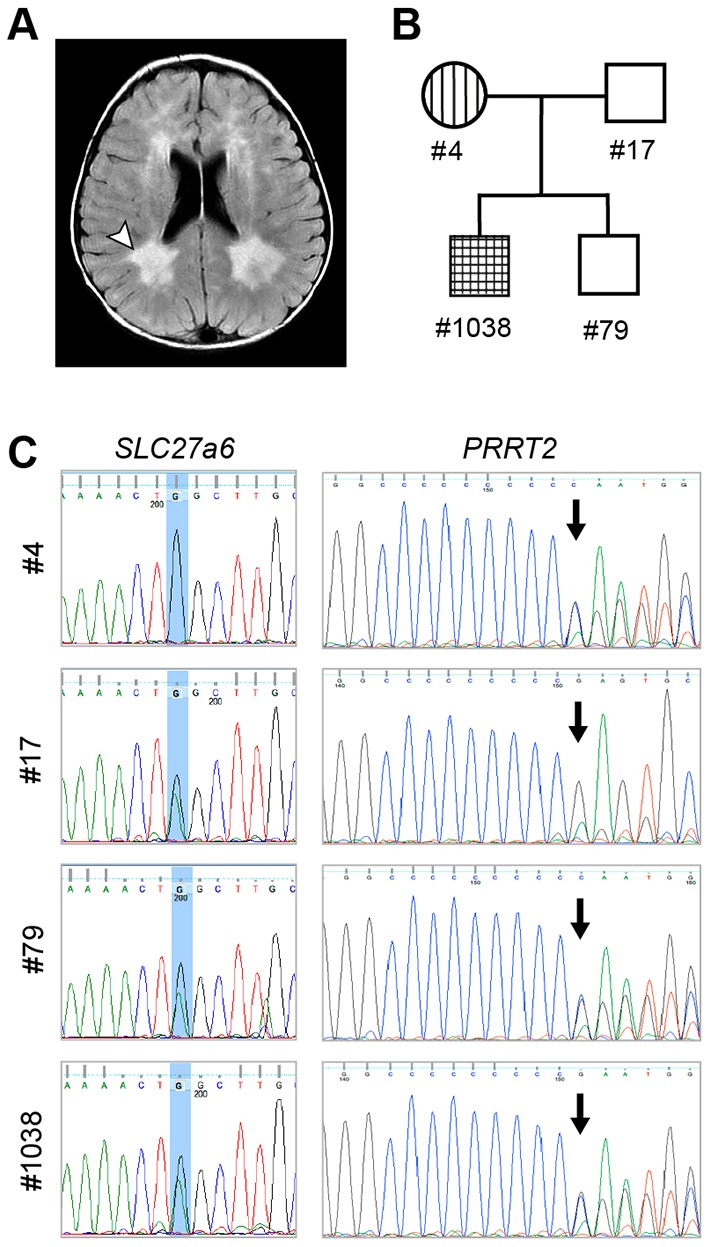
**Identification of a leukodystrophy patient with mutations in *SLC27a6*.** (A) MRI scan of an 18-month-old boy presenting with epilepsy. Arrowhead points to one of the expanded lateral ventricles, diagnostic of leukodystrophy; the paired structural defect is evident on the right side as well. (B) Pedigree shows family-member phenotypes: PRRT2-dependent benign familial infantile epilepsy (vertical hatching); leukodystrophy (horizontal hatching). The proband is designated as #1038. (C) Sequence data for *SLC27a6* and *PRRT2*, respectively. Carriers of the *SLC27a6* mutant allele have a C>T transition; the resultant nonsense mutation leads to premature termination of the protein. Carriers of the *PRRT2* mutant allele have a C duplication at position 649; this frameshift leads to premature termination of the protein.

Dysregulation of fatty acid metabolism is associated with X-ALD, but other than *ABCD1* no genes in the fatty acid metabolism pathway have been linked to human leukodystrophies (Gordon et al., 2014). In the care of a child with a magnetic resonance imaging (MRI)-based diagnosis of leukodystrophy (Fig. 7A,B), whole-exome sequencing revealed 44 genes harboring nonsense mutations. One of these genes, *SLC27a6*, is highly expressed in the heart, brain and adrenal glands (Uhlen et al., 2015), and encodes a 619-amino-acid very-long-chain ACS belonging to the FATP (fatty acid transport protein) lineage of the ACS superfamily (Fig. S4A). The FATP ACSs are additionally defined by their dual function as fatty acid transporters (Steinberg et al., 1999a, 2000). Intriguingly, FATP members of the ACS superfamily are found in every branch, suggesting that the ancestral form of the gene was dual functioning and that transport function was lost independently on at least four different occasions. Notably, the *Drosophila* ortholog of *SLC27a6*, *fatp*, is closely related to *bgm* and has been reported to be required for neuronal health and maintenance (Dourlen et al., 2012). Sequence comparisons indicate that neither Bgm nor Dbb functions as a fatty acid transport protein, and homology between Fatp and Bgm/Dbb is limited to the ACS domains (Fig. S4B).

The young Utah leukodystrophy patient is heterozygous for a C>T transition that leads to production of a truncated SLC27a6 protein 294 amino acids in length. This allele (rs183450464; here designated *SLC27a6^294^*) is very rare in the human population, having an overall allele frequency of 0.001% across all ethnicities [Exome Aggregation Consortium (ExAC); http://exac.broadinstitute.org, accessed 8th July 2015]. Sequence analysis of the Utah affected family indicates that both the proband and his brother inherited the *SLC27a6^294^* allele from their father; thus, if causative of leukodystrophy, mutation of *SLC27a6* leads to an incompletely penetrant leukodystrophy (and, as for *ABCD1*, another member of the fatty acid metabolic pathway, disease risk is enhanced by additional genetic and/or environmental factors). Although still correlative, our discovery that loss of *SLC27a6* ACS function is associated with leukodystrophy is highly provocative based on ACS loss-of-function studies in the fly reported here and elsewhere (Dourlen et al., 2012), and of high potential significance given clinicians' frequent inability to recognize the heritable basis of leukodystrophy, a debilitating and oftentimes lethal condition of childhood (Gordon et al., 2014).

Further comparative genotype analysis indicates that both the proband and his older brother also inherited a mutated *PRRT2* allele from their mother (Fig. 7C). Mutation of *PRRT2* leads to an incompletely penetrant dominant form of epilepsy (Labate et al., 2012; Steinlein et al., 2012). With respect to phenotypes, whereas the proband suffers from seizures and leukodystrophy, his brother is asymptomatic for both conditions (Fig. 7B). Family history interviews reveal that the proband's mother (*PRRT2^+/−^*) suffered from childhood seizures; his father's (*SLC27a6^+/−^*) CNS health status, however, remains unclear. Comparative genotype/phenotype data, coupled with our observation that treatment of the proband with levetiracetam to control seizures unexpectedly stemmed the progression of his neurodegenerative condition, leads us to speculate that, in this patient, seizures are the trigger of a neurodegenerative condition linked to a susceptibility locus, with *SLC27a6* being a likely candidate. There is ample evidence for other ALD cases where events such as epileptic seizure or traumatic brain injury have been proposed to precipitate onset of leukodystrophy symptoms in previously asymptomatic individuals (Weller et al., 1992; Raymond et al., 2010; Vawter-Lee et al., 2015).

## DISCUSSION

That mutations in lipid metabolic enzymes are associated with a variety of neurometabolic diseases, including adrenoleukodystrophy, Niemann-Pick disease, Fabry disease, Farber's disease, Tay Sachs disease and Zellweger syndrome, shows the nervous system to be highly sensitive to alterations in lipid homeostasis. This sensitivity is thought to result from the high metabolic demand of neurons and their supporting cells – a demand not shared by cells of other organ systems. Investigators have identified the genetic basis for some of these disorders, and a number of *Drosophila* models have been described (Min and Benzer, 1999; Huang et al., 2007; Phillips et al., 2008; Mast et al., 2011; Nakayama et al., 2011).

Here, we present the first fully penetrant genetic model of ALD. Building on the Min and Benzer model (Min and Benzer, 1999), we have shown that the *bgm^1^ dbb^1^* mutant fly exhibits gross neurodegenerative phenotypes in the optic lobe and retina. We also show that amorphic mutations in *bgm* and *dbb* lead to VLCFA accumulation and behavioral abnormalities. The consequences of the double knockout in the fly are profound, notably recapitulating essential features of human ALD, which were not obvious in the single mutants, and thereby providing the best evidence to date of a powerful animal model of how dysregulation of fatty acid metabolism can lead to neurodegeneration. In focusing on the similarities between fly and human phenotypes associated with VLCFA accumulation, our data suggest that ALD affects both neurons and their supporting cells, and that excess fatty acid accumulation is associated with widespread cell losses in the fly brain and to neuronal loss via lytic cell death. Our detection of inclusions in *bgm* *dbb* mutant flies augurs an attractive diagnostic tool, and the *bgm dbb* fly itself is expected to provide an effective genetic tool for the identification of drugs resolving the symptoms of ALD and neurodegenerative disease associated with accumulating VLCFAs. Finally, our identification of a leukodystrophy patient harboring a mutation in a gene with ACSL activity provides a basis for examination of this gene as a susceptibility factor in ALD.

### Neurodegeneration in ACS mutants

Although viable, *bgm* and *dbb* single and double mutants suffer from optic lobe and retinal degeneration. That the highest levels of neurodegeneration (both with respect to expressivity and penetrance) are observed in double mutants points to redundant functions for *bgm* and *dbb* in the *Drosophila* CNS (see Fig. 3). Functional overlap of *bgm* and *dbb* as long- and very-long-chain ACSs is corroborated by elevation of VLCFAs in the double-mutant flies but not in the single mutants (see Fig. 6A,B), as well as by the overlapping long-chain substrate specificities documented after heterologous expression in yeast (see Fig. 1D).

One of the most striking morphologic abnormalities in the *bgm^1^ dbb^1^* double mutants is the loss of pigment cells (see Fig. 4E,F). Pigment cell organization is integral to the fly retina's lattice-like appearance, and thus loss of these cells is sufficient to account for the gross defects in ommatidial organization that we observe in mutants. Analogous disordered ommatidial phenotypes have been described in fly models of Parkinson's neurodegenerative disease and in retinal degenerative disease (Batterham et al., 1996; Feany and Bender, 2000; Shamloula et al., 2002). In the case of the latter, retinal architectural disorder was associated with defects in pigment cells, as also shown in our model. Pigment cell loss is also sufficient to account for the fenestrated membrane dysmorphologies that we observed in mutants (see Figs 3H and 4A-D), because the fenestrated membrane is formed from the feet of pigment (and cone) cells. Based on these studies, it seems that the effects of defective fatty acid metabolism in the fly retina are far-reaching, perhaps affecting cell viability in both an autonomous and non-autonomous fashion, although this remains to be tested. These observations contribute to the increasing recognition that pigment cells, and glial cells in general, provide critical support to the nervous system (Nave, 2010).

We also documented disarray in laminal regions of the fly CNS, and our data indicate that cell loss is central to this defect as well. Monopolar neurons in *bgm^1^ dbb^1^* brains, although maintaining an intact nuclear morphology, show signs of extensive cell swelling and cytoplasmic clearing (see Fig. 5E-G). These ultrastructural features are characteristic of lytic cell death and of the pathological changes distinguishing lipid-rich oligodendrocyte degeneration during the initial stages of X-ALD pathogenesis (Ito et al., 2001; Tschape et al., 2002). In this regard, cerebral forms of X-ALD are associated with progressive inflammatory demyelination of white matter that is most evident in parieto-ocipital regions (Moser et al., 2007). This reaction is characterized by brain infiltration with cytotoxic T cells and macrophages, as well as elevation of inflammatory cytokines such as TNFα and interleukin I (Powers et al., 1992). Consistent with our characterization of monopolar neuron morphology in flies, brain autopsies in deceased individuals with ALD reveal loss of myelin and oligodendrocyte glial cells resulting from cytolytic, rather than programmed, cell death (Ito et al., 2001).

### Accumulating VLCFAs in ACSL mutants

A long-standing debate in the ALD field centers on the question of whether neurodegeneration results from an accumulation of saturated or unsaturated fatty acids. Like others, we have shown that ACSL mutation increases VLCFA levels; in *bgm dbb* double mutants, it is C24:1 and C26:1 levels that are elevated in a significant fashion (see Fig. 6A,B). With respect to human disease, our results substantiate: (1) reports of elevated unsaturated fatty acids (C24:1 and C26:1) in plasma and fibroblasts from individuals with X-ALD (Moser et al., 1980, 1999), and (2) suggestions that dietary monoene therapies such as ‘Lorenzo's oil’ are potentially inefficacious in the treatment of X-ALD (Sandhir et al., 1998). Together, these data contradict the commonly held view that accumulated saturated VLCFAs are causative of ALD. Even more provocative is the observation that, although all individuals harboring mutant alleles of *ABCD1* exhibit increased circulating VLCFA levels, many do not manifest any neurodegenerative symptoms (Steinberg et al., 2015; Dubey et al., 2005).

At the cellular level, accumulating granular material in the proximity of dying neurons (see Fig. 5F-H) points to the possibility that monopolar neuron death is due, at least in part, to toxicity of accumulated metabolic byproducts, even though Nile red and Sudan black stains suggest it is unlikely that *bgm dbb* laminal inclusions themselves represent fatty acid accumulations. Bolstering this alternative view are published studies, including: (1) the suggestion that excess fatty acids are causative of cell death in both Leydig and adrenocortical cells in X-ALD patients (Powers and Schaumburg, 1981), (2) evidence in cultured cells that free VLCFAs induce apoptosis by activation of the proinflammatory TNF-mediated pathway and by formation of free radicals that induce mitochondrial damage (Feigenbaum et al., 2000; Ulloth et al., 2003; Reiser et al., 2006), and (3) the demonstration in cultured rat hippocampal cells that VLCFA challenge specifically promotes their death (Hein et al., 2008). In *in vivo* models, however, although metabolic abnormalities are causative of cell death, accumulated lipids serve primarily as markers of neurodegeneration (Liu et al., 2015).

At the very least, inclusions in brain tissue and elevated levels of VLCFAs in *bgm dbb* mutant flies provide clear diagnostic markers, and point to the *Drosophila bgm dbb* mutant as a powerful *in vivo* model for testing the effects of fatty acid dysregulation on neurodegeneration. Moreover, our data suggest that the mechanisms of neuronal cell damage observed in *Drosophila bgm dbb* mutants are similar to those associated with the first stage of X-ALD pathogenesis. The primary assault on CNS cells in individuals with X-ALD is believed to result from impaired homeostasis of VLCFAs that leads to membrane instability, cell lysis and eventually to a fulminant inflammatory response (Powers et al., 2005).

### *Drosophila* models of neurodegeneration

The *Drosophila bgm dbb* mutant represents a valuable addition to a growing family of fly models of neurometabolic disease. Within this group, the neurodegenerative diseases have a variety of associated abnormalities. One of the best characterized in *Drosophila* is *swiss cheese* (*sws*; Muhlig-Versen et al., 2005; Dutta et al., 2016). *sws* mutants suffer from a *bgm dbb-*like age-dependent neurodegeneration, although holes develop more rapidly, more prominently, and across a wider expanse of the CNS than occurs in *bgm* and *dbb* mutants, and although the retina is notably spared. *sws* codes for an esterase, which, when disrupted in humans, leads to the neurodegenerative condition spastic paraplegia (Rainier et al., 2008). This esterase is required for hydrolyzing phosphatidylcholine, and loss-of-function mutations in either humans or flies results in an abnormal build up of phosphatidylcholine (Glynn, 2005; Muhlig-Versen et al., 2005; Fernandez-Murray and McMaster, 2007). Thus, the etiology of degeneration for neuropathy-target-esterase-mediated neurodegeneration is very likely analogous to that which is proposed for adrenoleukodystrophy – blockage in a metabolic pathway leading to an abnormal accumulation of lipids (Read et al., 2009).

More recent descriptions of *Drosophila* neurodegeneration mutants reveal those with age-dependent neurodegenerative phenotypes that, like *bgm* and *dbb*, target the retina. Intriguingly, two of these [*dPPCS* and *fumble* (also known as *dpank*)] function in the CoA synthetic pathway (Bosveld et al., 2008; Siudeja et al., 2011). The *Drosophila* model of neurometabolic disease most analogous to our *bgm/dbb* model is the *fatp* mutant. Animals harboring retinal clones homozygous for the null allele, *fatp^k10307^*, manifest age-dependent photoreceptor degeneration and additionally display disorganization of the retina (Dourlen et al., 2012, 2015). Although loss of pigment granule in *fatp* clones shown by Dourlen et al. is due to the loss of *w^+^* in mutant clones, it is also possible that the *fatp* mutation itself leads to a loss of pigment cells [although this is difficult to judge in a *w^–^* background (Pellikka et al., 2002)]. Nonetheless, it is intriguing to speculate on the potential for shared loss-of-function phenotypes among the long- and very-long-chain ACSs in *Drosophila* (*bgm*, *dbb* and *fatp*) and the possibility that these genes, and the lipid metabolic pathway(s) in which they act, play an essential role in neuromaintenance. Moreover, in the case of Fatp, shared loss-of-function phenotypes point to loss of synthetase function (rather than transporter function) as causative of neurodegeneration; however, this remains to be tested. Finally, in light of the specific *bgm*, *dbb* and *fatp* shared loss-of-function phenotypes in flies, it is noteworthy that our patient study reveals a correlation of *SLC27a6*, the human *fatp* ortholog, with leukodystrophy in humans.

### Association of a human ACSL with leukodystrophy

Our identification of the first leukodystrophy patient to harbor a nonsense mutation in *SLC27a6*, a gene encoding a predicted dual-functioning ACSL and fatty acid transporter, is particularly compelling because it suggests that mutations in the ACSL family of genes can be causative of neurodegeneration in humans, as they are in flies. Indeed, it has long been thought that ACSL mutations might be associated with ALD because ACSL levels are decreased in individuals with ALD (Hashmi et al., 1986; Lazo et al., 1988; Wanders et al., 1988). The mode of inheritance of *SLC27a6^294^*-associated neurodegenerative disease seems to be autosomal dominant, as is the case for several well-documented neurodegenerative conditions in humans (Bertram and Tanzi, 2005). The nonsense mutation that we document in our leukodystrophy patient is quite rare (allele frequency: 0.001335), albeit more common than any of the other 41 predicted loss-of-function alleles in the ExAC database (allele frequency: 0.00007120), with the exception of one (allele frequency: 0.0022) (ExAC; http://exac.broadinstitute.org, accessed 8th July 2015). The two most common alleles are almost certainly nulls: one with premature termination after amino acid 294; the other with a frameshift after amino acid 8. It is likely they each arose only once in isolated populations because each clusters in a single ethnic population database. We expect that homozygosity for loss-of-function *SLC27a6* alleles results in a highly penetrant zygotic lethality, because only one loss-of-function homozygote (for any allele) has been identified to date. The scarcity of mutant alleles in the human population, coupled with a paucity of diagnostic sequence data in affected individuals and an incompletely penetrant phenotype, provides an explanation for the failure (until now) to associate *SLC27a6* with leukodystrophy. Thus, like fly *bgm* and *dbb*, and human *ABCD1*, *SLC27a6* might also be causative of an incompletely penetrant neurodegenerative phenotype, albeit in dominant fashion.

### Summary

The *bgm dbb* double-mutant animal has provided unique insights into the organismal (locomotor activity), tissue (neurodegeneration) and cellular (monopolar neuron and pigment cell death) requirements for long- and very-long-chain fatty acids. CNS degeneration that is associated with behavioral and biochemical abnormalities in both flies and humans underscores the power of this model for continuing to probe disease etiology as well as to initiate investigation of palliative treatment options. Moreover, in addition to revealing severe neurological involvement, activity phenotypes can be monitored in higher-throughput assays, and this opportunity underscores the feasibility of suppression screens for the identification of compounds and/or dietary regimens that alleviate the abnormalities associated with ACS mutation. Finally, highlighting the continuing power of simple invertebrate genetic models, this study has led to our recognition of a new candidate susceptibility gene for catastrophic presentation of leukodystrophy (Vawter-Lee et al., 2015).

## MATERIALS AND METHODS

### Fly stocks

Balancers, Canton-S, *w1118* and *Df(bgm)* {*Df(2L)b87e25/CyO* [34B12-C1;35B10- C1]} have been described (The FlyBase Consortium, 2003). *bgm^1^* was from Kyung-Tai Min (NINDS). Strains for targeted mutagenesis were from Kent Golic (University of Utah).

### Targeted mutagenesis

Ends-out gene targeting was performed as described (Gong and Golic, 2003). Our targeting construct was engineered to delete the 5′ two-thirds of the *dbb* coding region. To this end, DNA fragments corresponding to 14023363-14026942 and 14028337-14031358 in the *Drosophila melanogaster* genome were subcloned into the pW25 plasmid. Targeting was verified in progeny by PCR (using 5′-CGAAAGGGAGAGTTCGACTCC-3′ and 5′-GGCTGCTGCAATAAGATTAGGGC-3′ primers) and by RT-PCR (using 5′-CGGATATCACACTGTCAC-3′ and 5′-CATCGGCATACATCTTGGACA-3′ for *bgm*, 5′-GCCATCATCCTTTTCACAGAC-3′ and 5′-GTACAGACGCTCGATGACTTTG-3′ for *dbb*, and 5′-GTGCCGAATACATCGTGGAG-3′ and 5′-AACGACCTCCTCATCGGTGT-3′ for *Gapdh2*).

### Yeast rescue

BY4743, a strain mutant for the yeast gene *FAA1* (the generous gift of Diane Ward, University of Utah), was transfected with constructs containing *Drosophila bgm* or *dbb* cDNAs cloned into the yeast expression vector p426-ADH1. Yeast cultures were grown on YPD (yeast peptone dextrose; standard growth medium) supplemented with cerulenin (Cayman Chemical, Ann Arbor, MI) to inhibit *de novo* synthesis. Oleate and myristate were obtained from Nu Check Prep (Elysian, MN). Geneticin (G418) selection was employed to monitor the *FAA1* mutant background and 5-fluoroorotic acid (5-FOA) was employed to monitor the p426-ADH1 vector.

### Activity and lifespan studies

Wild-type and mutant flies were maintained in a 12 h:12 h light:dark cycle at 25°C. For activity phenotyping, 6-18 males/genotype were individually transferred to monitor tubes on the day of eclosion, and to fresh tubes weekly. Locomotor activity was monitored using the *Drosophila* Activity Monitoring System starting within 24 h of eclosion and continuing each week until day 30 post-eclosion (Trikinetics, Waltham, MA). Average peak activity was determined by analyzing beam break occurrences during the 2 h before and after light change after acclimatization into the activity chambers. For longevity studies, 40-100 flies/genotype were seeded at an initial density of 20 flies/vial. Survivors were transferred to fresh vials every 7 days. Curves are estimated from the results of three independent trials. GraphPad Prism software was employed for statistical analyses.

### Histology and transmission electron microscopy

For histological examination, heads were dissected and processed as described (Palladino et al., 2002). Serial 6-µm sections were stained with H&E, and scored blindly under a light microscope for the presence of vacuoles with diameter greater than 3 μm, retinal degeneration and fenestrated membrane changes. For TEM studies, heads were embedded in Spurr's epoxy resin, sectioned at 70-90 nm thickness using a Reichert-Jung Ultracut E microtome, post-fixed with osmium and stained with 2% uranyl acetate and Reynolds' lead citrate. Ultrastructural analysis was carried out using a Hitachi H-7100 electron microscope at 125 kV. For tangential sections shown in Fig. S2, adult *Drosophila* males were prepared by rinsing heads in methanol (MeOH) and fixing in 4% formaldehyde/PBS. Heads were dehydrated through serial MeOH/H_2_O washes and equilibrated in Solution A before embedding (100 ml Solution A:1 gram catalyst, Electron Microscopy Sciences Immuno-Bed Infiltrate cat. no. 14260-01, 14260-06, respectively). Embedding was performed by mixing 1:20 Solutions B:A (Solution B, Electron Microscopy Sciences cat. no. 14260-04). Plastic blocks were sectioned at 8 μm thickness and stained with 0.1% Toluidine blue to increase photoreceptor contrast. All samples were imaged at 600× using a Zeiss Axioskop 50 and Axiovision4 camera software.

### Fatty acid profiling

Extracts from 18-day-old males and females were used for purification and methylation of fatty acids. Solutions were analyzed by GC-MS using a Micromass GCT Premier time-of-flight mass spectrometer fitted with an Agilent 6890 gas chromatograph and autosampler (Waters Corp., Beverly, MA). Fatty acids increasing in chain length from C12 were assayed and a *P*-value less than 0.05 was used as a cut-off for significance.

### Oil red O stains

Dissected larval gut and cryosectioned adult heads were fixed in 10% formalin/PBS for 30 min, followed by one wash with water and two with 100% propylene glycol. Tissues were submerged in Oil red O, heated to 60°C for 15 min, destained in three 85% propylene glycol and one water wash, and mounted in 80% glycerol.

### Patient analysis

This study was approved by Institutional Review Boards of the University of Utah and Intermountain Healthcare (IH), and informed consent was obtained. Clinical exome sequencing was performed through Baylor Medical Genetics Laboratories. Confirmatory Sanger sequencing for *SLC27a6* was performed using primers (5′-3′): (forward) TGAGCAAACATGTTCTAGACAGAG and (reverse) TACCTTTGTTACTGGCGGGT. Template DNA was isolated from cheek cells in all individuals shown in the pedigree.
